# Supplementary material for: Cat and dog owners’ expectations and attitudes towards advanced veterinary care (AVC) in the UK, Austria and Denmark
Source: PLoS One. 2024 Mar 20;19(3):e0299315. doi: 10.1371/journal.pone.0299315 (PMC10954172; doi:10.1371/journal.pone.0299315)
Supplement: S2 File — (DOCX) [file pone.0299315.s002.docx]

| **Statement 2. AUSTRIA. My pet should have access to the same treatment options that are available to human patients**  The ordinal regression analysis was statistically significant (LR-χ^2^(9)=280.287, P=0.000, Nagelkerke’s R^2^=0.312) | | | | | |
| --- | --- | --- | --- | --- | --- |
|  |  |  | **Hypothesis Test** | | |
|  | **B** | **Std. Error** | **Wald Chi-Square** | **df** | **Sig** |
| Gender | -0.064 | 0.1565 | 0.165 | 1 | 0.685 |
| Live alone or not | 0.212 | 0.1645 | 1.658 | 1 | 0.198 |
| Pet Health Insurance | -0.042 | 0.1701 | 0.061 | 1 | 0.805 |
| Species (dog or cat) | -0.030 | 0.1476 | 0.041 | 1 | 0.840 |
| Income (prefer not to say/I don’t know) | -0.044 | 0.2082 | 0.045 | 1 | 0.833 |
| Income (low) | 0.177 | 0.1939 | 0.829 | 1 | 0.362 |
| Income (middle) | 0.082 | 0.1791 | 0.212 | 1 | 0.645 |
| Age | 0.002 | 0.0043 | 0.130 | 1 | 0.719 |
| LAPS mean | 2.460 | 0.1634 | 226.754 | 1 | 0.000 |
| **Statement 2. DENMARK. My pet should have access to the same treatment options that are available to human patients**  The ordinal regression analysis was statistically significant (LRχ^2^(9)=233.443, P=0.000, Nagelkerke’s R^2^=0.322) | | | | | |
|  |  |  | **Hypothesis Test** | | |
|  | **B** | **Std. Error** | **Wald Chi-Square** | **df** | **Sig** |
| Gender (M or F) | -0.024 | 0.1635 | 0.021 | 1 | 0.885 |
| Live alone or not | 0.066 | 0.2077 | 0.100 | 1 | 0.752 |
| Insurance (yes or no) | 0.491 | 0.1618 | 9.218 | 1 | 0.002 |
| Species (dog or cat) | 0.226 | 0.1632 | 1.912 | 1 | 0.167 |
| Income (low) | 0.448 | 0.2302 | 3.785 | 1 | 0.052 |
| Income (middle) | 0.474 | 0.2308 | 4.217 | 1 | 0.040 |
| Income (high) | 0.525 | 0.1901 | 7.615 | 1 | 0.006 |
| Age | -0.007 | 0.0048 | 2.468 | 1 | 0.116 |
| LAPS mean | 2.032 | 0.1726 | 138.574 | 1 | 0.000 |
| **Statement 2. UK. My pet should have access to the same treatment options that are available to human patients**  The ordinal regression analysis was statistically significant (LRχ^2^(9)=179.714, P=0.000, Nagelkerke’s R^2^=0.244) | | | | | |
|  |  |  | **Hypothesis Test** | | |
|  | **B** | **Std. Error** | **Wald Chi-Square** | **df** | **Sig** |
| Gender | -0.333 | 0.1579 | 4.448 | 1 | 0.035 |
| Live alone or not | -0.132 | 0.1874 | 0.497 | 1 | 0.481 |
| Insurance | 0.212 | 0.1492 | 2.014 | 1 | 0.156 |
| Species (dog or cat) | 0.019 | 0.1546 | 0.015 | 1 | 0.902 |
| Income (low) | -0.439 | 0.2619 | 2.806 | 1 | 0.094 |
| Income (middle) | -0.293 | 0.1941 | 2.272 | 1 | 0.132 |
| Income (high) | -0.442 | 0.1830 | 5.827 | 1 | 0.016 |
| Age | 0.003 | 0.0044 | 0.452 | 1 | 0.501 |
| LAPS mean | 1.956 | 0.1647 | 141.112 | 1 | 0.000 |
| **Statement 3. AUSTRIA. I would enrol my pet in a research study to help advance veterinary care as long as the risk of potential complications was low**  The ordinal regression analysis was statistically significant (LRχ^2^(9)=69.453, P<0.001, Nagelkerke’s R^2^=0.087) | | | | | |
|  |  |  | **Hypothesis Test** | | |
|  | **B** | **Std. Error** | **Wald Chi-Square** | **df** | **Sig** |
| Gender (M or F) | 0.262 | 0.1521 | 2.962 | 1 | 0.085 |
| Live alone or not | -0.003 | 0.1602 | 0.000 | 1 | 0.985 |
| Insurance (yes or no) | 0.579 | 0.1642 | 12.420 | 1 | <0.001 |
| Species (dog or cat) | -0.123 | 0.1435 | 0.730 | 1 | 0.393 |
| Income (low) | -0.403 | 0.2053 | 3.850 | 1 | 0.050 |
| Income (middle) | -0.228 | 0.1866 | 1.494 | 1 | 0.222 |
| Income (high) | 0.025 | 0.1744 | 0.020 | 1 | 0.886 |
| Age | -0.011 | 0.0041 | 7.601 | 1 | 0.006 |
| LAPS mean | 0.812 | 0.1432 | 32.104 | 1 | <0.001 |
| **Statement 3. DENMARK. I would enrol my pet in a research study to help advance veterinary care as long as the risk of potential complications was low**  The ordinal regression analysis was statistically significant (LRχ^2^(9)=31.687 P<0.001, Nagelkerke’s R^2^=0.051) | | | | | |
|  |  |  | **Hypothesis Test** | | |
|  | **B** | **Std. Error** | **Wald Chi-Square** | **df** | **Sig** |
| Gender (M or F) | 0.268 | 0.1596 | 2.823 | 1 | 0.093 |
| Live alone or not | 0.158 | 0.2023 | 0.611 | 1 | 0.434 |
| Insurance (yes or no) | 0.048 | 0.1589 | 0.093 | 1 | 0.761 |
| Species (dog or cat) | 0.068 | 0.1597 | 0.183 | 1 | 0.669 |
| Income (low) | -0.304 | 0.2259 | 1.815 | 1 | 0.178 |
| Income (middle) | -0.083 | 0.2252 | 0.135 | 1 | 0.714 |
| Income (high) | -0.072 | 0.1855 | 0.151 | 1 | 0.697 |
| Age | -0.015 | 0.0048 | 9.495 | 1 | 0.002 |
| LAPS mean | 0.585 | 0.155 | 14.162 | 1 | <0.001 |
| **Statement 3. UK. I would enrol my pet in a research study to help advance veterinary care as long as the risk of potential complications was low**  The ordinal regression analysis was statistically significant (LRχ^2^(9)=64.671 P<0.001, Nagelkerke’s R^2^=0.094) | | | | | |
|  |  |  | **Hypothesis Test** | | |
|  | **B** | **Std. Error** | **Wald Chi-Square** | **df** | **Sig** |
| Gender | 0.157 | 0.1529 | 1.061 | 1 | 0.303 |
| Live alone or not | 0.199 | 0.1777 | 1.248 | 1 | 0.264 |
| Insurance | 0.063 | 0.1460 | 0.186 | 1 | 0.666 |
| Species (dog or cat) | 0.010 | 0.1526 | 0.004 | 1 | 0.949 |
| Income (low) | -0.603 | 0.2569 | 5.501 | 1 | 0.019 |
| Income (middle) | -0.453 | 0.1887 | 5.759 | 1 | 0.016 |
| Income (high) | -0.697 | 0.1793 | 15.132 | 1 | <0.001 |
| Age | -0.016 | 0.0044 | 13.082 | 1 | <0.001 |
| LAPS mean | 0.783 | 0.1475 | 28.177 | 1 | <0.001 |
| **Statement 6. AUSTRIA. The advanced care available in modern veterinary medicine has gone ‘too far’ putting animals through ‘too much’**  The ordinal regression analysis was statistically significant (LRχ^2^(9)=25.639, P=0.002, Nagelkerke’s R^2^=0.034) | | | | | |
|  |  |  | **Hypothesis Test** | | |
|  | **B** | **Std. Error** | **Wald Chi-Square** | **df** | **Sig** |
| Gender (M or F) | 0.280 | 0.1555 | 3.251 | 1 | 0.071 |
| Live alone or not | -0.046 | 0.1632 | 0.080 | 1 | 0.777 |
| Insurance (yes or no) | 0.578 | 0.1774 | 10.628 | 1 | 0.001 |
| Species (dog or cat) | -0.111 | 0.1468 | 0.571 | 1 | 0.450 |
| Income (low) | 0.376 | 0.2089 | 3.235 | 1 | 0.072 |
| Income (middle) | 0.164 | 0.1940 | 0.713 | 1 | 0.399 |
| Income (high) | 0.140 | 0.1791 | 0.613 | 1 | 0.434 |
| Age | -0.001 | 0.0043 | 0.086 | 1 | 0.769 |
| LAPS mean | 0.363 | 0.1444 | 6.330 | 1 | 0.012 |
| **Statement 6. DENMARK. The advanced care available in modern veterinary medicine has gone ‘too far’ putting animals through ‘too much’**  The ordinal regression analysis was statistically significant (LRχ^2^(9)=18.895, P=0.026, Nagelkerke’s R^2^=0.032) | | | | | |
|  |  |  | **Hypothesis Test** | | |
|  | **B** | **Std. Error** | **Wald Chi-Square** | **df** | **Sig** |
| Gender (M or F) | -0.362 | 0.1669 | 4.692 | 1 | 0.030 |
| Live alone or not | -0.089 | 0.2124 | 0.176 | 1 | 0.675 |
| Insurance (yes or no) | -0.146 | 0.1648 | 0.784 | 1 | 0.376 |
| Species (dog or cat) | -0.135 | 0.1681 | 0.649 | 1 | 0.420 |
| Income (low) | 0.011 | 0.2388 | 0.002 | 1 | 0.962 |
| Income (middle) | -0.038 | 0.2392 | 0.025 | 1 | 0.874 |
| Income (high) | 0.251 | 0.1958 | 1.645 | 1 | 0.200 |
| Age | 0.013 | 0.0049 | 7.375 | 1 | 0.007 |
| LAPS mean | -0.079 | 0.1583 | 0.252 | 1 | 0.616 |
| **Statement 6. UK. The advanced care available in modern veterinary medicine has gone ‘too far’ putting animals through ‘too much’**  The ordinal regression analysis was statistically significant (LRχ^2^(9)=38.499, P<0.001, Nagelkerke’s R^2^=0.058) | | | | | |
|  |  |  | **Hypothesis Test** | | |
|  | **B** | **Std. Error** | **Wald Chi-Square** | **df** | **Sig** |
| Gender | 0.122 | 0.1580 | 0.599 | 1 | 0.439 |
| Live alone or not | 0.380 | 0.1813 | 4.383 | 1 | 0.036 |
| Insurance | -0.089 | 0.1503 | 0.354 | 1 | 0.552 |
| Species (dog or cat) | 0.085 | 0.1565 | 0.294 | 1 | 0.588 |
| Income (low) | -0.167 | 0.2560 | 0.426 | 1 | 0.514 |
| Income (middle) | -0.296 | 0.1962 | 2.284 | 1 | 0.131 |
| Income (high) | -0.528 | 0.1840 | 8.237 | 1 | 0.004 |
| Age | -0.021 | 0.0044 | 21.946 | 1 | <0.001 |
| LAPS mean | 0.228 | 0.1468 | 2.411 | 1 | 0.121 |
| **Statement 7. AUSTRIA The advanced care available in modern veterinary medicine is unnecessary – animals should not be treated in the same way as humans**  The ordinal regression analysis was statistically significant (LRχ^2^(9)=76.313, P<0.001, Nagelkerke’s R^2^=0.096) | | | | | |
|  |  |  | **Hypothesis Test** | | |
|  | **B** | **Std. Error** | **Wald Chi-Square** | **df** | **Sig** |
| Gender (M or F) | 0.689 | 0.1552 | 19.695 | 1 | <0.001 |
| Live alone or not | 0.014 | 0.1622 | 0.007 | 1 | 0.933 |
| Insurance (yes or no) | 0.591 | 0.1742 | 11.510 | 1 | <0.001 |
| Species (dog or cat) | -0.314 | 0.1438 | 4.775 | 1 | 0.029 |
| Income (low) | -0.019 | 0.2040 | 0.008 | 1 | 0.927 |
| Income (middle) | -0.161 | 0.1921 | 0.699 | 1 | 0.403 |
| Income (high) | -0.329 | 0.1745 | 3.546 | 1 | 0.060 |
| Age | -0.008 | 0.0043 | 3.102 | 1 | 0.078 |
| LAPS mean | -0.618 | 0.1442 | 18.338 | 1 | <0.001 |
| **Statement 7. DENMARK The advanced care available in modern veterinary medicine is unnecessary – animals should not be treated in the same way as humans**  The ordinal regression analysis was statistically significant (LRχ^2^(9)=83.210, P<0.001, Nagelkerke’s R^2^=0.130) | | | | | |
|  |  |  | **Hypothesis Test** | | |
|  | **B** | **Std. Error** | **Wald Chi-Square** | **df** | **Sig** |
| Gender (M or F) | 0.016 | 0.1595 | 0.010 | 1 | 0.918 |
| Live alone or not | -0.178 | 0.2070 | 0.743 | 1 | 0.389 |
| Insurance (yes or no) | -0.233 | 0.1612 | 2.089 | 1 | 0.148 |
| Species (dog or cat) | -0.062 | 0.1613 | 0.150 | 1 | 0.699 |
| Income (low) | -0.211 | 0.2268 | 0.869 | 1 | 0.351 |
| Income (middle) | -0.106 | 0.2303 | 0.213 | 1 | 0.644 |
| Income (high) | 0.260 | 0.1904 | 1.864 | 1 | 0.172 |
| Age | 0.016 | 0.0048 | 11.157 | 1 | <0.001 |
| LAPS mean | -0.986 | 0.1597 | 38.134 | 1 | <0.001 |
| **Statement 7. UK The advanced care available in modern veterinary medicine is unnecessary – animals should not be treated in the same way as humans**  The ordinal regression analysis was statistically significant (LRχ^2^(9)=72.592, P<0.001, Nagelkerke’s R^2^=0.105) | | | | | |
|  |  |  | **Hypothesis Test** | | |
|  | **B** | **Std. Error** | **Wald Chi-Square** | **df** | **Sig** |
| Gender | 0.503 | 0.1545 | 10.603 | 1 | 0.001 |
| Live alone or not | 0.465 | 0.1759 | 6.991 | 1 | 0.008 |
| Insurance | -0.093 | 0.1460 | 0.404 | 1 | 0.525 |
| Species (dog or cat) | -0.049 | 0.1517 | 0.105 | 1 | 0.746 |
| Income (low) | -0.298 | 0.2509 | 1.415 | 1 | 0.234 |
| Income (middle) | -0.388 | 0.1909 | 4.120 | 1 | 0.042 |
| Income (high) | -0.616 | 0.1785 | 11.897 | 1 | <0.001 |
| Age | -0.025 | 0.0043 | 32.708 | 1 | <0.001 |
| LAPS mean | -0.267 | 0.1424 | 3.508 | 1 | 0.061 |
