# Supplementary material for: Cat and dog owners’ expectations and attitudes towards advanced veterinary care (AVC) in the UK, Austria and Denmark
Source: PLoS One. 2024 Mar 20;19(3):e0299315. doi: 10.1371/journal.pone.0299315 (PMC10954172; doi:10.1371/journal.pone.0299315)
Supplement: S3 File — (DOCX) [file pone.0299315.s003.docx]

| **Practice type attended with the DOG(S)** | | | | | | |
| --- | --- | --- | --- | --- | --- | --- |
|  | | **All**  **(N=1245)** | **Austria**  **(n=409)** | **Denmark**  **(n=385)** | **UK**  **(n=451)** |  |
| Small animal practice  (1-3 vets) | 679 (54.7) | | 287 (71.7) | 224 (58.6) | 168 (36.9) | χ^2^(2)=74,905, p<0.001  AT vs DK: p<0.001  AT vs UK: p<0.001  DK vs UK:  p<0.001 |
| Small animal practice  (4 or more vets) | 299 (23.9) | | 51 (11.8) | 103 (26.2) | 145 (32.5) |  |
| Mixed practice | 99 (8.4) | | 4 (1.2) | 26 (6.8) | 69 (15.9) |  |
| University hospital | 42 (3.4) | | 24 (5.8) | 1 (0.2) | 17 (4.0) |  |
| Charity clinic | 16 (1.2) | | 3 (0.7) | 1 (0.3) | 12 (2.4) |  |
| Several types of practice | 37 (2.7) | | 23 (5.1) | 7 (1.9) | 7 (1.2) |  |
| I do not have a veterinary practice that I attend | 32 (1.5) | | 10 (2.1) | 6 (1.5) | 16 (3.7) |  |
| I don't know | 41 (3.1) | | 7 (1.6) | 17 (4.4) | 17 (3.5) |  |
| **Practice type attended with the CAT(S)** | | | | | | |
|  | **All**  **(N=1273)** | | **Austria**  **(n=575)** | **Denmark**  **(n=318)** | **UK**  **(n=380)** |  |
| Small animal practice  (1-3 vets) | 730 (65.5) | | 399 (69.0) | 186 (57.9) | 145 (37.4) | χ^2^(2)=141.142, p<0.001  AT vs DK: p<0.001  AT vs UK: p<0.001  DK vs UK:  p<0.001 |
| Small animal practice  (4 or more vets) | 244 (19.9) | | 55 (9.8) | 63 (19.9) | 126 (34.7) |  |
| Mixed practice | 67 (5.3) | | 8 (1.3) | 16 (5.0) | 43 (11.3) |  |
| University hospital | 56 (4.7) | | 44 (8.4) | 4 (1.3) | 8 (2.2) |  |
| Charity clinic | 25 (1.9) | | 5 (0.8) | 8 (2.4) | 12 (3.2) |  |
| Several types of practice | 24 (1.7) | | 15 (2.2) | 6 (1.9) | 3 (0.9) |  |
| I do not have a veterinary practice that I attend | 86 (6.8) | | 38 (6.8) | 20 (6.9) | 28 (6.9) |  |
| I don't know | 41 (3.0) | | 11 (1.7) | 15 (4.7) | 15 (3.5) |  |

**Supporting Information 3.**

**Type of practice usually attended by owners of dog(s) and / or cat(s).**

*Number of respondents (n) and analyses with inferential statistics were calculated with unweighted data; Proportions were calculated with weighted data; rounding errors lead to some differences between rounded-off numerical values and actual values*
