# Supplementary material for: Cat and dog owners’ expectations and attitudes towards advanced veterinary care (AVC) in the UK, Austria and Denmark
Source: PLoS One. 2024 Mar 20;19(3):e0299315. doi: 10.1371/journal.pone.0299315 (PMC10954172; doi:10.1371/journal.pone.0299315)

**Supporting Information 4.**

**Pearson Correlation analysis to explore whether owners who believe their pets should have access to advanced veterinary care also likely to think that their vets should contribute knowledge to the advancement of such care, and be willing to enrol their own pets ino a research study.**


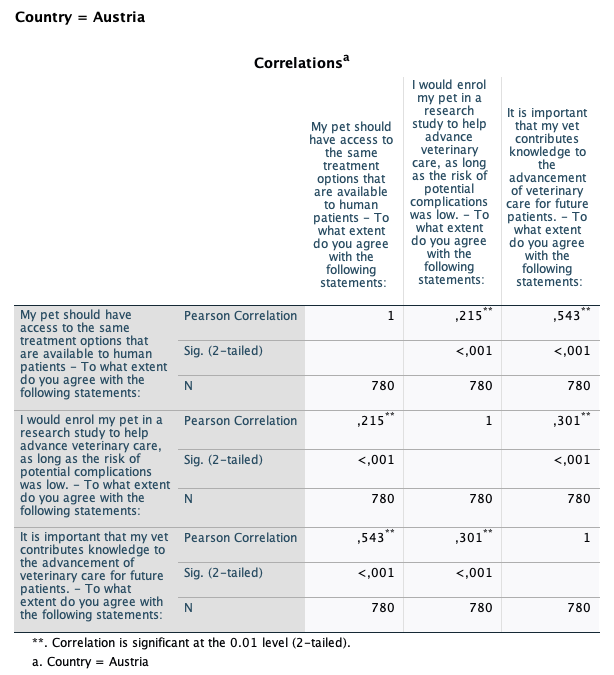


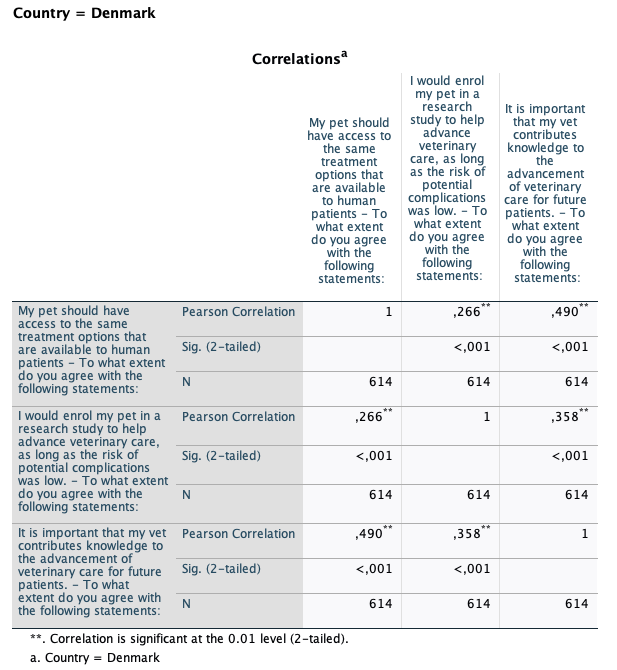


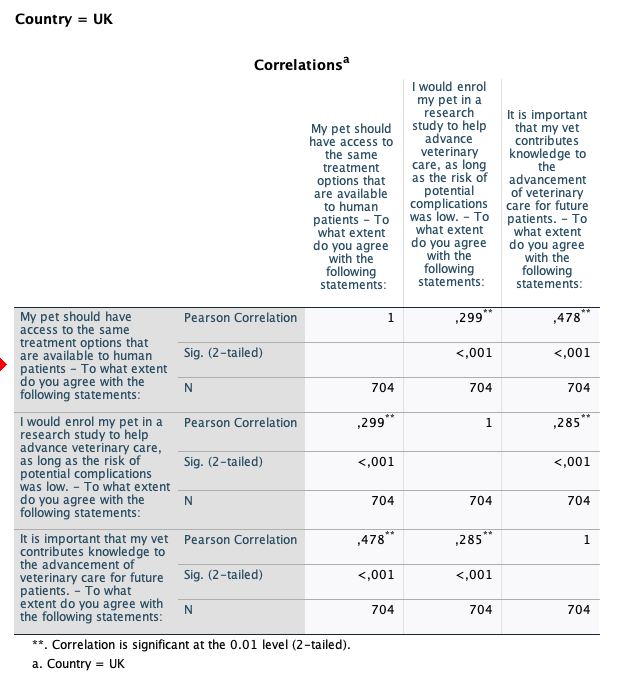

Supplement: S4 File — (DOCX) [file pone.0299315.s004.docx]
