# Supplementary material for: Cat and dog owners’ expectations and attitudes towards advanced veterinary care (AVC) in the UK, Austria and Denmark
Source: PLoS One. 2024 Mar 20;19(3):e0299315. doi: 10.1371/journal.pone.0299315 (PMC10954172; doi:10.1371/journal.pone.0299315)
Supplement: S5 File — (DOCX) [file pone.0299315.s005.docx]

**Supporting Information 5. Differences in uptake of pet health insurance (yes/ no, not anymore) and income (low, middle, high |excluded I don’t know/prefer not to say): Kruskal Wallis H Test.**

**Austria**: H(1)=0.014, p=0.906

**Denmark**: H(1)=3.269, p=0.071

**UK**: H(1)=31.472, p<0.001

| **Health insurance YES - Income UK grouped^a^** | | | | | |
| --- | --- | --- | --- | --- | --- |
|  | **Income** | **Frequency** | **Percent** | **Valid Percent** | **Cumulative Percent** |
| Valid | **LOW** (less than 11200-22399 GBP) | 67 | 7.6 | 20.7 | 20.7 |
|  | **MIDDLE** (22400-44799 GBP) | 122 | 13.9 | 37.7 | 58.3 |
|  | **HIGH** (44800-more than 112000 GBP) | 135 | 15.4 | 41.7 | 100.0 |
|  | **Total** | 324 | 37.0 | 100.0 |  |
| Missing | **I don’t know / prefer not to say** | 31 | 3.5 |  |  |
|  | **System** | 521 | 59.5 |  |  |
|  | **Total** | 552 | 63.0 |  |  |
| Total |  | 876 | 100.0 |  |  |

^a^ Health Insurance Binary = yes

| **Health Insurance NO / NOT ANYMORE – Income UK grouped^a^** | | | | | |
| --- | --- | --- | --- | --- | --- |
|  | **Income** | **Frequency** | **Percent** | **Valid Percent** | **Cumulative Percent** |
| Valid | **LOW** (less than 11200-22399 GBP) | 120 | 9.7 | 40.4 | 40.4 |
|  | **MIDDLE** (22400-44799 GBP) | 102 | 8.2 | 34.3 | 74.7 |
|  | **HIGH** (44800-more than 112000 GBP) | 75 | 6.0 | 25.3 | 100.0 |
|  | **Total** | 297 | 23.9 | 100.0 |  |
| Missing | **I don’t know / prefer not to say** | 39 | 3.1 |  |  |
|  | **System** | 905 | 72.9 |  |  |
|  | **Total** | 944 | 76.1 |  |  |
| Total |  | 1241 | 100 |  |  |

^a^ Health Insurance Binary = no / not anymore
