# Supplementary material for: Cat and dog owners’ expectations and attitudes towards advanced veterinary care (AVC) in the UK, Austria and Denmark
Source: PLoS One. 2024 Mar 20;19(3):e0299315. doi: 10.1371/journal.pone.0299315 (PMC10954172; doi:10.1371/journal.pone.0299315)
Supplement: S6 File — (DOCX) [file pone.0299315.s006.docx]

**Supporting Information 6. Correlations between LAPS scores and age, gender, living status, insurance and income.**

**1. LAPS and AGE: Pearson Correlation.**

| **ALL** | | **Current Age** | **LAPS Mean** |
| --- | --- | --- | --- |
| Current Age | Pearson Correlation | 1 | -0.041 |
|  | Sig. (2-tailed) |  | 0.062 |
|  | N | 2116 | 2116 |
| LAPS Mean | Pearson Correlation | -0.41 | 1 |
|  | Sig. (2-tailed) | 0.062 |  |
|  | N | 2116 | 2117 |
| **AUSTRIA** | | **Current Age** | **LAPS Mean** |
| Current Age | Pearson Correlation | 1 | 0.025 |
|  | Sig. (2-tailed) |  | 0.485 |
|  | N | 800 | 800 |
| LAPS Mean | Pearson Correlation | 0.025 | 1 |
|  | Sig. (2-tailed) | 0.485 |  |
|  | N | 800 | 800 |
| DENMARK | | Current Age | LAPS Mean |
| Current Age | Pearson Correlation | 1 | -0.060 |
|  | Sig. (2-tailed) |  | 0.134 |
|  | N | 625 | 625 |
| LAPS Mean | Pearson Correlation | -0.060 | 1 |
|  | Sig. (2-tailed) | 0.134 |  |
|  | N | 625 | 626 |
| UK | | Current Age | LAPS Mean |
| Current Age | Pearson Correlation | 1 | -0.041 |
|  | Sig. (2-tailed) |  | 0.280 |
|  | N | 691 | 691 |
| LAPS Mean | Pearson Correlation | -0.041 | 1 |
|  | Sig. (2-tailed) | 0.280 |  |
|  | N | 691 | 691 |

**2. LAPS and GENDER, LIVING STATUS and INSURANCE: Mann-Whitney-U-Test**

H(1)=95507.000; p<0.001 for the Mann Whitney U Test.

| **LAPS and GENDER** | | | |
| --- | --- | --- | --- |
| **Country** | **Number** | **H (1)** | **P value** |
| All | 2109 | 658041.500 | 0.000 |
| Austria | 796 | 95507.000 | <0.001 |
| Denmark | 625 | 57685.500 | <0.001 |
| UK | 688 | 70393.500 | <0.001 |
| **LAPS and LIVING STATUS** | | | |
| **Country** | **Number** | **H (1)** | **P value** |
| All | 2117 | 327725.500 | <0.001 |
| Austria | 800 | 50656.000 | 0.136 |
| Denmark | 626 | 23525.500 | <0.001 |
| UK | 691 | 36419.000 | 0.739 |
| **LAPS and INSURANCE** | | | |
| **Country** | **Number** | **H (1)** | **P value** |
| All | 2117 | 480841.000 | <0.001 |
| Austria | 800 | 48050.000 | 0.032 |
| Denmark | 626 | 34298.000 | <0.001 |
| UK | 691 | 52704 | 0.008 |

**3. PAIRWISE COMPARISONS OF INCOME GROUPS WITH LAPS SCORES FOR EACH COUNTRY: Independent-Samples Kruskal-Wallis Test**

| **AUSTRIA** | Test Statistic | Standard Error | Std. Test Statistic | Significance | Adjusted significance^a^ |
| --- | --- | --- | --- | --- | --- |
| **HIGH** (53800 - more than 134500 EURO)  **MIDDLE** (26900 - 53799 EUR0) | 35.441 | 18.316 | 1.935 | 0.053 | 0.159 |
| **HIGH** (53800 - more than 134500 EURO)  **LOW** (less than 13450 - 26899 EURO) | 86.000 | 18.808 | 4.573 | <0.001 | 0.000 |
| **MIDDLE** (26900 - 53799 EURO)  **LOW** (less than 13450 - 26899 EURO) | 50.560 | 17.775 | 2.844 | 0.004 | 0.013 |
| **DENMARK** | Test Statistic | Standard Error | Std. Test Statistic | Significance | Adjusted significance^a^ |
| **HIGH** (600.001 - more than 1.000.001 DK)  **MIDDLE** (300.003 - 600.000 DK) | 21.758 | 15.581 | 1.396 | 0.163 | 0.488 |
| **HIGH** (600.001 - more than 1.000.001 DK)  **LOW** (less than 100.000 - 300.000DK) | 86.609 | 16.702 | 5.186 | <0.001 | 0.000 |
| **MIDDLE** (300.003 - 600.000 DK)  **LOW** (less than 100.000 - 300.000DK) | 64.851 | 17.494 | 3.707 | <0.001 | 0.001 |
| **UK** | Test Statistic | Standard Error | Std. Test Statistic | Significance | Adjusted significance^a^ |
| **HIGH** (44800 - more than 112000 GBP)  **MIDDLE** (22400 - 44799 GBP) | 13.208 | 17.224 | 0.767 | 0.443 | 1.000 |
| **HIGH** (44800 - more than 112000 GBP)  **LOW** (Less than 11200 - 22399 GBP) | 32.940 | 18.029 | 1.827 | 0.068 | 0.203 |
| **MIDDLE** (22400 - 44799 GBP)  **LOW** (Less than 11200 - 22399 GBP) | 19.732 | 17.762 | 1.111 | 0.267 | 0.800 |

Each row tests the null hypothesis that the Sample 1 and Sample 2 distributions are the same.
Asymptotic significances (2-sided tests) are displayed. The significance level is 0.050.

a. Significance values have been adjusted by the Bonferroni correction for multiple tests.
